# Does Adiponectin Act as an Antiangiogenic Factor in B-Cell Chronic Lymphocytic Leukemia?

**DOI:** 10.1155/2009/287974

**Published:** 2009-08-27

**Authors:** Stefano Molica, Giovanna Digiesi, Angelo Vacca, Rosanna Mirabelli, Katia Todoerti, Caterina Battaglia, Fortunato Morabito, Antonino Neri, Domenico Ribatti

**Affiliations:** ^1^Medical Oncology Unit, Department of Hematology-Oncology, Azienda Ospedaliera Pugliese-Ciaccio, 88100 Catanzaro, Italy; ^2^Department of Clinical Pathology, IRCCS Regina Elena, Roma, Italy; ^3^Department of Internal Medicine and Clinical Oncology, University of Bari Medical School, Bari, Italy; ^4^Laboratorio di Genetica Molecolare, IRCCS Policlinico, Milano, Italy; ^5^Department of Hematology, Azienda Ospedaliera Verona, Cosenza, Italy; ^6^Department of Human Anatomy and Histology, University of Bari Medical School, Bari, Italy

## Abstract

Angiogenesis is involved in the pathogenesis of B-cell chronic lymphocytic leukemia (CLL), and high microvascular density has been found in CLL to be associated with a poor prognosis. In this study, we assessed serum levels of adiponectin in 69 patients with Binet stage A B-CLL, and these values were retrospectively correlated with bone marrow (BM) microvessel area and serum levels of vascular endothelial growth factor (VEGF), fibroblast growth factor-2 (FGF-2), angiogenin, PECAM-1 (CD31), matrix metalloproteinase-9 (MMP-9), interleukin-8 (IL-8), syndecan-1, and the percentage of CD38^+^ or ZAP-70^+^ CLL cells. The positive correlation between serum levels of adiponectin and VEGF (*P* = .03) does not translate into an increase of the extent of BM angiogenesis (*P* = .404), FGF-2 (*P* = .348), angiogenin (*P* = .402), and CD31 (*P* = .248) serum concentrations. Accordingly, IL-8 (*P* = .175), syndecan-1 (*P* = .06), and MMP-9 (*P* = .144) circulating levels were not likely to reflect adiponectin concentration. Furthermore, patients with higher levels of adiponectin had a more favorable biological profile as defined by a lower number of both CD38^−^ (*r* = −0.294; *P* = .02) and ZAP-70^+^ (*r* = −0.285; *P* = .04). Finally, we evaluated the presence of adiponectin in B-CLL cells at gene expression level. RMA intensity values for adiponectin gene transcript denote a homogeneous low expression in B-CLL cells, whereas VEGF transcript was highly expressed with a degree of interpatient variability. Overall, these data seem to indicate that adiponectin could be involved as an antiangiogenic factor in B-CLL.

## 1. Introduction

Angiogenesis is involved in the pathogenesis of B cell chronic lymphocytic leukemia (CLL), and high microvascular density has been found in CLL to be associated with a poor prognosis [1]. Several angiogenic growth factors are involved in the pathogenesis of CLL. Serum vascular endothelial growth factor (VEGF) and fibroblast growth factor-2 (FGF-2) were elevated in CLL patients, and high VEGF levels also predicted disease progression in early stage CLL [2–4]. A study including 216 CLL patients showed that those with higher concentration of VEGF receptor-2 (VEGFR-2) in their peripheral blood lymphocytes had significantly shorter survival [5]. Angiogenin was expressed in CLL cells and in B cells of normal donors, although its expression is higher in B-cells CLL leukemic cells in comparison to normal B cells [6]. Frater et al. [7] demonstrated an upregulation of hypoxia inducible factor-1 alpha (HIF-1*α*) in CLL, suggesting that localized tissue hypoxia is another important activator of microvessel proliferation in CLL. Finally, matrix metalloproteinases (MMPs) have important roles in angiogenesis and Bauvois et al. [8] found significantly higher levels of MMP-9 in patients with CLL than in healthy controls.

Adiponectin, an adipose tissue-derived peptide, is secreted by adipose cells and mimics many metabolic actions of insulin [9]. Moreover, adiponectin is a regulator of energy homeostasis and plays a role in the obesity-induced insulin resistance and related complications [10].

More recently, it has been established a role for adiponectin in the homeostasis of the cardiovascular system. Adiponectin activated adenosine monophosphate kinase (AMP-K) in endothelial cells, leading to enhanced in vivo angiogenesis in murine Matrigel plug and rabbit cornea assays, and inhibition of caspase 3-mediated apoptosis in endothelial cells cultured in vitro [11, 12]. Otherwise, Bråkenhielm et al. [13] showed that adiponectin inhibited endothelial cell migration and proliferation in vitro and neoangiogenesis in vivo in the chick embryo chorioallantoic membrane (CAM) and cornea assays as well as decreased angiogenesis and induced apoptosis in tumors.

With the aim to investigate the involvement of adiponectin in angiogenesis in B-CLL, we have assessed serum levels of adiponectin in 69 patients with Binet stage A CLL, and these values were retrospectively correlated with bone marrow microvessels area and serum levels of VEGF, FGF-2, angiogenin, PECAM-1 (CD31), MMP-9, interleukin-8 (IL-8), syndecan-1, and the percentage of CD38^+^ or ZAP-70^+^ CLL cells. Finally, we have evaluated the presence of adiponectin and VEGF in B-CLL cells at gene expression levels.

## 2. Materials and Methods

### 2.1. CLL Patients' Cohorts

Two independent series of previously untreated Binet stage A CLL patients were investigated. The first series included 69 patients followed at the Medical Oncology Department of Catanzaro; the second cohort included 60 Binet stage A CLL patients from a cooperative database specifically devised to investigate the gene expression profiling of CLL cells.

### 2.2. Characteristics of Patients Evaluated for Serum Adiponectin Level

Sixty-nine patients were selected based on the availability of frozen serum samples. Their median age was 64 years (range, 40–83), and the male to female ratio was 41 to 28.

### 2.3. Measurement of Adiponectin and Other Cytokines

Serum adiponectin concentrations were measured in duplicate with a quantitative sandwich enzyme immunoassay (ELISA) technique (Human Adiponectin Quantikine Colorimetric ELISA, R & D Systems, Minneapolis, Minn, USA). Calibrations were carried out with human adiponectin standards. Optical densities at 450 nm were measured with a microtiter plate reader. A standard curve was created by plotting the logarithm of the mean absorbance of each standard versus the logarithm of the cytokine concentration. Concentrations were expressed in *μ*g/mL, and the coefficient of variation (CV) reported by the manifacturer for interassay and intraassay determinations ranged from 6.9% to 8.8% and from 2.5% to 4.7%, respectively. Serum vascular endothelial growth factor (VEGF), FGF-2, angiogenin, PECAM-1 (CD31), MMP-9, and IL-8 levels were measured with a quantitative ELISA (Quantikine; R & D Systems, Minneapolis, Minn, USA). Serum syndecan-1 concentrations were determined as serum immune reactivity using a quantitative sandwich enzyme immunoassay (ELISA) technique (syndecan-1 ELISA, Diaclone Research, Besancon, France).

### 2.4. Measurement of BM Angiogenesis

All blood vessels were displayed in 6-*μ*m sections of 4% paraformaldehyde-fixed paraffin embedded biopsies by staining endothelial cells with the antifactor VIII-related antigen murine monoclonal antibody M616 (IgG1; Dako, Glostrup, Denmark) and a three-layer biotin-streptavidin peroxidase system described previously [3]. The very few megakaryocytes also stained with factor VIII-related antigen were easily distinguishable by their morphology and size. Microvessel counts were assessed in (4–6) × 250 fields per section (covering almost the whole section) and 2 sections for biopsy within a superimposed 484-point square reticle of 12.5 × 10^−2^ mm^2^/field. Microvessels (i.e., capillaries and small venules) were identified as tubes with a single layer of endothelial cells, either without or with a lumen not exceeding 10 *μ*m. A planimetric point-count method was applied to count only those microvessels transversely sectioned which occupied the reticle intersection points.

### 2.5. Detection of ZAP-70

Mononuclear cells (MNCs) from CLL cases were isolated by Ficoll-Hypaque (Seromed, Biochrom KG, Berlin, Germany) density gradient centrifugation, stained with CD19-PE and CD3 PE-CY7 (Becton Dickinson, San Diego, Calif, USA), and fixed and permeabilized with fix & perm reagents (Caltag Laboratories, Burlingame, Calif, USA) at the concentration of 5 × 10^5^ cells/50 *μ*L. The cells were subsequently exposed to ZAP-70 mAb conjugated with FITC (clone 2F3.2, Upstate, lake Placid, NY, USA) and analyzed by flow cytometry (FacsCalibur Becton Dickinson). A CD33 FITC (Becton Dikinson) mAb of the same IgG1 subclass was used as negative control of ZAP-70 [14].

### 2.6. CD-38 Evaluation

Direct immunofluorescent staining was performed with CD19 FITC/PE, CD23 PE, CD38 PE, and Cy-Chrome (Becton Dickinson & Co., Sunnyvale, Calif, USA). CD19 FITC, CD23 PE, and CD5 Cy-Chrome were used to assess the proportion of CLL cells in the suspensions; the proportion of CD38-positive leukemic cells was determined by triple staining for CD19 FITC, CD38 PE, and CD5 Cy-Chrome. The cells were analyzed using a FACS-sort flow cytometer (Becton Dickinson & Co).

### 2.7. Gene Expression

T cells, NK cells, and monocytes were removed from PBMNC of CLL patients by CD3, CD56, CD16, and CD14 monoclonal antibody (mAb) treatment (Becton Dickinson, San Diego, Calif, USA) followed by magnetic beads (Goat Anti-Mouse IgG D ynabeads, Dynal Biotech ASA, Oslo, Norway). The purity of the B cells was assessed by flow cytometry by the determination of the proportions of CD5/CD19/CD23 triple positive B cells in the suspension. Purified normal peripheral blood B cells were obtained from six normal donors. Total RNA was isolated using the TRIzol Reagent (Life Technologies, Inc., Rockville, Md, USA) and then purified using the RNeasy total RNA Isolation Kit (Qiagen, Valencia, Calif, USA). Preparation of biotin-labeled cRNA, hybridization to GeneChip Human Genome U133A Arrays (Affymetrix Inc., Santa Clara, Calif, USA) and scanning of the chips (7G Scanner, Affymetrix Inc., Santa Clara, Calif, USA) were carried out according to manufacturer's protocols. The images were analysed using Affymetrix GeneChip Operating Software (GCOS) 1.4, and the probe level data were converted to expression values using the Bioconductor function for Robust Multiarray average (RMA) procedure, in which perfect match values are background adjusted and normalized using quantile-quantile normalization, as previously described [14]. Expression data of the 60 B-CLLs proprietary database were deposited in the National Centre for Biotechnology Information's Gene Expression Omnibus (GEO; http://www.ncbi.nlm.nih.gov/geo/) and are available through GEO Series accession number GSE11038

### 2.8. Statistics

Spearman correlations, Mann-Whitney test, and the corrected 2c test were applied to compare groups.

## 3. Results

### 3.1. Adiponectin Concentration in CLL Patients

Adiponectin serum levels in Binet stage A patients ranged between 1.1 and 22.0 *μ*g/mL (median, 5.88 *μ*g/mL). In healthy age- and sex-matched controls the median was 5.4 *μ*g/mL (range, 1.2–11.8 *μ*g/mL). Overall, no difference could be found between patients with CLL and healthy controls (Mann-Whitney test, *P* = .658). Interestingly either normal controls or CLL patients had a body mass index (BMI) <30 Kg/m^2^.

### 3.2. Correlation between Adiponectin and Other Angiogenic Factors

**Figure 1:**
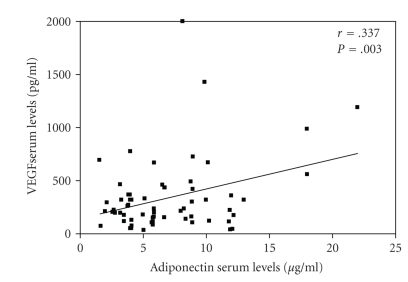
Correlation between serum levels of adiponectin and VEGF. The analysis denotes a significant direct correlation (*r* = 0.337; *P* = .003).

In order to assess the potential angiogenic of adiponectin in CLL we analyzed its correlation with other angiogenic factors. Serum levels of adiponectin reflected VEGF concentration (*r* = 0.337; *P* = .03; see Figure 1); however, such an association did not translate into an increase of the extent of bone marrow angiogenesis in patients with higher adiponectin levels. In 27 patients with available data we failed to demonstrate any association between microvessel area and adiponectin (*P* = .404). A similar behavior was observed for FGF-2 (*P* = .348), angiogenin (*P* = .402), and CD31 (*P* = .248) whose serum concentrations did not change as a function of adiponectin levels. Accordingly, IL-8 (*P* = .175), syndecan-1 (*P* = .06), and MMP-9 (*P* = .144) circulating levels were not likely to reflect adiponectin concentrations.

### 3.3. Adiponectin and Biologic Profile in CLL

**Figure 2:**
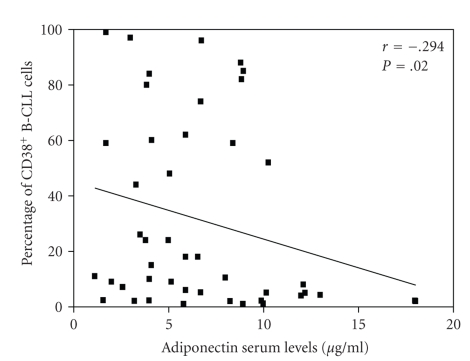
Correlation between serum levels of adiponectin and percentage of CD38-positive B-CLL cells. The analysis denotes a significant inverse correlation (*r* = −0.294; *P* = .02).

Given the transcriptional characteristics of CD38^+^ cells in CLL whose VEGF level is 2-3 fold higher in comparison to CD38^−^ cells [15], we sought for correlation between CD38-expression and adiponectin concentration. The low angiogenic activity of adiponectin was also reflected in the inverse correlation between CD38 and adiponectin (*r* = −0.294; *P* = .02; see Figure 2). Furthermore, patients with higher levels of adiponectin had a more favorable biological profile as defined by a lower number of ZAP-70^+^ cells (*r* = −0.285; *P* = .04).

### 3.4. Evaluation of Adiponectin and VEGF Gene Transcript in CLL

**Figure 3:**
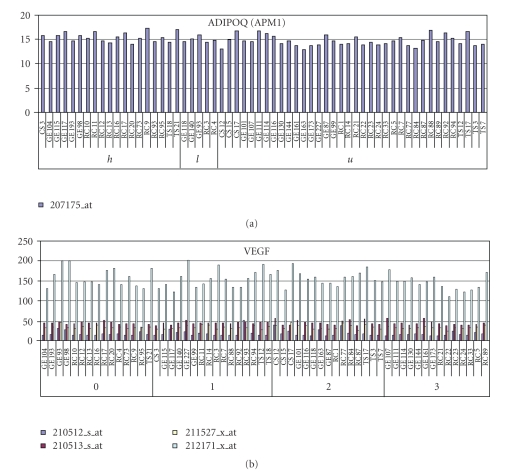
Absolute RMA intensity values for adiponectin (ADIOQ) (a) and VEGF (b) transcript in 60 B-CLL patients assessed by microarray analysis.

We examinated the presence of adiponectin in B-CLL cells at gene expression levels. RMA intensity values for adiponectin gene transcript denote a homogeneous low expression in B-CLL cells (Figure 3(a)). The same does not apply for VEGF transcript which was highly expressed with a degree of interpatients variability (Figure 3(b)).

## 4. Discussion

In this study we failed to demonstrate a proangiogenic role for adiponectin in B-CLL. As a matter of fact, the positive correlation between adiponectin and VEGF serum levels observed in such patients does not translate into an increase of other parameters involved in angiogenesis, such as BM microvessel area, serum concentrations of FGF-2, angiogenin, IL-8, syndecan-1, CD31, and MMP-9.

We previously demonstrated an association between increased serum VEGF concentration and modern prognostic factors in B-cell CLL [16]. Using high-speed cell sorting and subsequent gene expression profiling, Pepper et al. [15] investigated the transcriptional characteristics of purified CD38^+^ and CD38^−^ subsets from a CLL patient and demonstrated 2-3 fold increased levels of VEGF in CD38^+^ cells. The inverse correlation between circulating levels of adiponectin and either CD38 or ZAP-70 expression, observed in this study, suggests that B-CLL patients with high-serum adiponectin levels may have a less aggressive phenotype.

Finally, evaluating adiponectin and VEGF gene transcripts, we have demonstrated a homogeneous low expression of adiponectin gene transcripts in B-CLL cells, whereas VEGF transcripts were highly expressed with a degree of interpatient variability. These results suggest that leukemic cells have a limited role in the production of circulating adiponectin levels. On the contrary both adiponectin receptor 1 (AdipoR1) and AdipoR2 mRNA were highly expressed by CLL cells [14]. Whether the effect of adiponectin on cell proliferation might be most likely adiponectin receptor-mediated also in CLL as in other models is not clear so far [17].

Angiogenesis is controlled by the balance between molecules that have positive and negative regulatory activity and this concept led to the notion of angiogenic switch, which depends on an increased production of one or more positive regulators of angiogenesis [18]. Endothelial cell turnover in the healthy adult organism is low, the quiescence being maintained by the dominant influence of endogenous angiogenesis inhibitors over angiogenic stimuli. In pathological situations angiogenesis may be triggered not only by the overproduction of proangiogenic factors, but also by the downregulation of inhibitory factors. Also in B-CLL, the balance of pro- and antiangiogenic factors appears to have a critical role in its pathogenesis. Kay et al. [19] indicated that B-CLL cells expresssed pro- and antiangiogenic molecules, and Shanafelt and Kay [20] observed, that the ratio of proangiogenic FGF-2 and antiangiogenic thrombospondin-1 (TSP-1) correlated with the time to treatment. Ligation of TSP-1 receptor appears to induce a caspase-independen apoptotic death in CLL B-cells, which occurs even if these cells are cocultured with autologous marrow stromal cells or fibroblasts, therefore suggesting that the angiogenic switch is related to clinical progression in B-cell CLL.

The data presented in this study seem to indicate that adiponectin could be involved as an antiangiogenic factor in B-cell CLL. Our data are in keeping with those published by Bora et al. [21], who have demonstrated that adiponectin acts as an antiangiogenic protein in the mouse model of laser-induced choroidal neovascularization and inhibits the expression of VEGF mRNA and protein in the choroidal tissue. Finally, Mahadev et al. [22] demonstrated that adiponectin suppressed VEGF-stimulated human coronary endothelial cells migration via cAMP/PKA-dependent signalling, with important implications for a regulatory role of adiponectin in vascular processes associated with diabetes and atherosclerosis.

It is well established that the angiogenic phenotype results from the imbalance between positive and negative regulator factors, so that the contribution of each “classical” and/or “nonclassical” angiogenic factor may play a different role in the definition of the angiogenic phenotype [23]. In this context, adiponectin can be considered as one of the “nonclassical” angiogenic factors. A detailed knowledge of the mechanism of action and expression as well as the interactions of the new “non-classical” endogenous regulators of angiogenesis with their receptors will provide new insights that are essential for the future development of chemical compounds that can modulate the activity of these new “non-classical” endogenous regulators and may have potential for antitumor activity. In fact, tumors and other angiogenic pathologies exploit redundant mechanisms to induce angiogenesis, and neutralization of mutiple factors, including both “classical” and “nonclassical” regulators may be required to suppress tumor growth. The recognition by clinicians of the angiogenesis-modulatory properties of common drugs is important in the development and future application of angiogenesis inhibitory therapies.
